# Experimental study on the effect of CNT-enriched nanofluid lubrication on the performance of textured cutting tool in the turning of aluminum 7075 alloy

**DOI:** 10.1038/s41598-023-48796-w

**Published:** 2023-12-19

**Authors:** Salman Khani

**Affiliations:** Mechanical Engineering Department, Amirkabir University of Technology, Tehran, Iran

**Keywords:** Mechanical engineering, Engineering, Materials science

## Abstract

This paper investigates the impact of surface texturing and the use of CNT-enriched nanofluid lubrication on the cutting performance of cemented carbide cutting tools during the turning process of aluminum 7075 alloy. Aluminum 7075 is widely utilized in various industries due to its exceptional properties, including high corrosion resistance, a favorable strength-to-weight ratio, and good formability. However, this alloy tends to excessively adhere to the cutting tool at the tool-chip interface, which negatively affects the machining process. Previous research has proposed different solutions, but the current study focuses on implementing the two most effective approaches to minimize adhesion phenomena. The first approach involves modifying the contact area by creating a pattern on the tool's rake face, while the second approach utilizes CNT-enriched nanofluid lubrication to reduce friction in the tool-chip interface. Various types of surface textures were fabricated on the rake face, and experimental tests were conducted to identify the most effective texture. The findings showed that using textured tools with micro-grooves perpendicular to the chip flow direction, with CNT-enriched nanofluid lubrication, resulted in significant reductions in main cutting force, built-up edge, and surface finish. The decreases were up to 32%, 37%, and 19%, respectively, compared to dry turning conditions.

## Introduction

The remarkable properties of aluminum alloys, such as their impressive strength and stiffness-to-weight ratio, high corrosion resistance, excellent electrical and heat conductivity, and favorable formability, make them widely utilized in industries such as machinery manufacturing, aerospace, marine, and automobile^1^. While aluminum alloys possess favorable cutting properties attributed to their low strength, they tend to exhibit adhesion between the tool and chip on the rake face during machining operations^2^. Traditionally, cutting fluids have been employed to mitigate friction and adhesion in the machining process. However, the use of cutting fluids poses risks to machine operators, leads to damage to machine tool rails, and contributes to environmental pollution. In light of these concerns, there is a growing demand to minimize or eliminate the use of cutting fluids and transition towards dry machining methods that align with environmentally friendly manufacturing processes. To address these concerns, optimization studies have been conducted^3, 4^ and various sustainable approaches have been adopted in manufacturing to promote greener and cleaner production and mitigate the limitations associated with dry machining^5–7^.

Some of the methods employed to address the challenges associated with cutting fluids include the development of new tool materials, optimization of cutting fluid application through techniques like minimum quantity lubrication machining^8^, cryogenic machining^9^, and hybrid machining^10^, as well as the modification of traditional cutting fluid properties using nanofluids^11^, and the modification of cutting tool surfaces through approaches like surface texturing^12, 13^.

In material removal processes, traditional cutting fluids have been employed to lubricate the interfaces between the tool and chip as well as the tool and workpiece, dissipate heat generated within the cutting zone, and facilitate the removal of chips from the machining area. The utilization of cutting fluids has resulted in enhanced productivity in manufacturing. However, the costs associated with cutting fluids and the health risks associated with exposure to cutting fluid mist have prompted researchers to explore approaches aimed at reducing or eliminating the reliance on these fluids. Some of these methods include the utilization of textured tools and the incorporation of nanofluids.

The application of surface texturing on cutting tools is acknowledged as a technique to improve the effectiveness of dry machining practices. This approach aims to enhance the tribological conditions between mating surfaces^14^. By introducing surface textures on cutting tools, the friction coefficient can be reduced through increased lubrication capacity and a decrease in the length of tool-chip contact. Researchers have successfully implemented various types of textures on cutting tool surfaces in drilling^15^, milling^16^, turning^17^, and thread turning^18–20^.

In a study conducted by Xie et al.^21^, a range of micro-grooves with depths ranging from 7 to 149 μm and aspect ratios between 0.14 and 0.5 were fabricated on the rake face of cutting tools using micro-grinding techniques. The results demonstrated that as the depth of the micro-grooves decreased, the cutting temperature decreased and the shear angle increased. Furthermore, their findings indicated that for improved cutting tool performance, it was necessary for the width of the grooves to be narrower than the chip width. In another study conducted by Fang and Obikawa^22^, five different types of micro-textures were created on the flank face of carbide inserts. These textures had depths/heights ranging from 10 to 20 μm and widths of 50 μm. The purpose of the micro-textures was to enhance the cooling effectiveness of high-pressure jet coolant during the turning of Inconel 718 alloy. The findings of the study demonstrated that the use of micro-textured tools resulted in reduced flank and crater wear compared to traditional tools. The tool with a micro-pit array with a depth of 10 μm exhibited the best performance, reducing flank wear by 50% compared to the plain tool. Dry turning experiments were performed by Liu et al.^23^ on green alumina using tools with textured flank faces, and the wear resistance was investigated. The micro-groove dimensions were set at a width of 35 μm, a depth of 20 μm, and a spacing of 200 μm between the grooves. The results revealed a significant reduction in flank face wear when using textured tools compared to conventional tools. Furthermore, it was observed that the micro-grooves on the flank face aligned parallel to the main cutting edge, exhibited superior resistance to flank wear. The researchers concluded that the mechanism responsible for the reduction in flank face wear was the derivative-cutting phenomenon occurring on the flank face. In a separate investigation conducted by Khani et al.^18, 20^ the effectiveness of textured tools filled with various solid lubricants was evaluated during the threading of Al 7075 alloy. The textured tools featured microholes with a depth, diameter, and pitch distance of 13, 70, and 200 μm, respectively. The study revealed that the use of a microhole textured tool filled with CNT (carbon nanotube) powder enhanced the performance of the thread-cutting process. The results indicated a reduction in cutting forces, built-up edge formation, and tool-chip contact length.

Tatiana et al.^24^ conducted a study to assess the performance of straight and zig–zag patterns created on the rake face of carbide tools using ultrashort laser pulses during the turning process of martensitic stainless steel. The research aimed to compare the performance of these patterns in terms of cutting forces, power consumption, and workpiece cylindricity deviation. The findings indicated that the straight pattern demonstrated superior performance compared to the zig–zag pattern across the evaluated parameters.

Nanofluids refer to a mixture of solid particles with nanometer-scale dimensions suspended in a conventional fluid, forming a solid–liquid composite^25^. Recently, nanofluids have gained prominence in various engineering applications, such as heat transfer systems and machining processes, owing to their exceptional heat transfer capabilities and tribological properties^25^. A review of the available literature reveals that the utilization of nanofluids in metal cutting processes enhances heat transfer, resulting in decreased cutting forces, cutting temperature, power consumption, and tool wear^26^.

Research has been conducted on cutting fluids based on carbon nanotubes (CNTs) to examine their impact on machining performance. For instance, Prabhu^27^ investigated the grinding of AISI D2 tool steel using dry conditions, conventional cutting fluid, and a nanofluid containing CNTs as a lubricant. The experimental results demonstrated that the utilization of a CNT-enriched nanofluid led to a significant improvement in surface finish, achieving a nano-level quality as opposed to a micro-level finish. In a separate investigation by the same author^28^, the addition of carbon nanotube particles to SAE20W40 oil was found to enhance the heat-absorbing capacity of the lubricant. The research findings demonstrated that the utilization of a cutting fluid based on carbon nanotubes resulted in improved surface finish and reduced occurrence of micro-cracks during the grinding process of D3 tool steel. Experimental research was conducted by Rao et al.^29^ to assess the cutting temperature and tool wear in the turning process. The results of the research indicated that the inclusion of CNT particles resulted in a reduction in nodal temperatures, leading to improved surface finish and increased tool life. The experimental results also indicated that the addition of CNT nanoparticles to conventional cutting fluid increased its thermal conductivity. Furthermore, it was observed that the thermal conductivity of the nanofluid increased with rising temperatures. These properties collectively highlighted the superior performance of CNT-based nanofluids in comparison to conventional coolants^30^. The application of multi-walled carbon nanotubes with minimum quantity lubrication (MQL) in hard turning of AISI H13 steel showcases the effectiveness and productivity of using nanofluid-MQL in conjunction with carbide tools for machining hot work tool steel in industrial applications^31, 32^.

Several research studies have focused on investigating the impact of surface texturing on the cutting performance of cemented carbide cutting tools. Also, there are several research works that investigated CNT-nanofluid application in machining processes^26^, however, there is a limited number of studies that have explored the combined effect of nanofluid lubrication and surface texturing. Consequently, there exists a research gap in this particular domain. In order to enhance the cutting performance of cemented carbide turning tools, this study proposes the implementation of micro-textured tools, along with CNT-enriched nanofluid lubrication, for the turning process of Aluminum 7075 alloy. Laser micromachining was employed to engrave four different types of textures, including linear and circular arrays, on the rake face of the carbide cutting inserts. Turning tests were subsequently conducted on Aluminum 7075 bars, utilizing both non-textured and textured tools, to determine the optimal texture. Subsequently, a comparative analysis was conducted by employing the selected textured tool under dry conditions and varying concentrations of CNT-enriched nanofluid lubrication.

## Materials and experimental procedures

**Figure 1:**
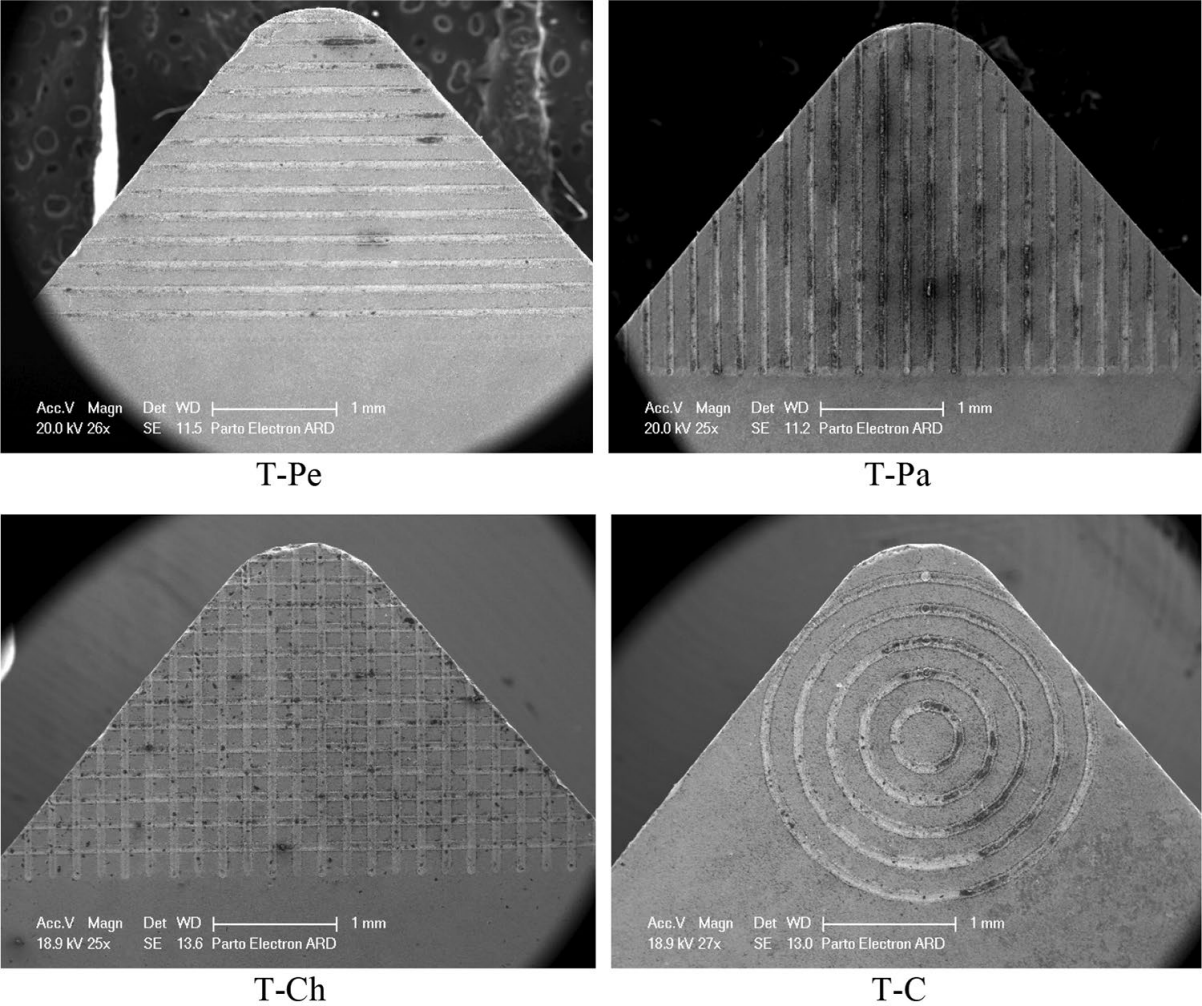
SEM images of microtextures generated at the rake face of carbide tools.

**Figure 2:**
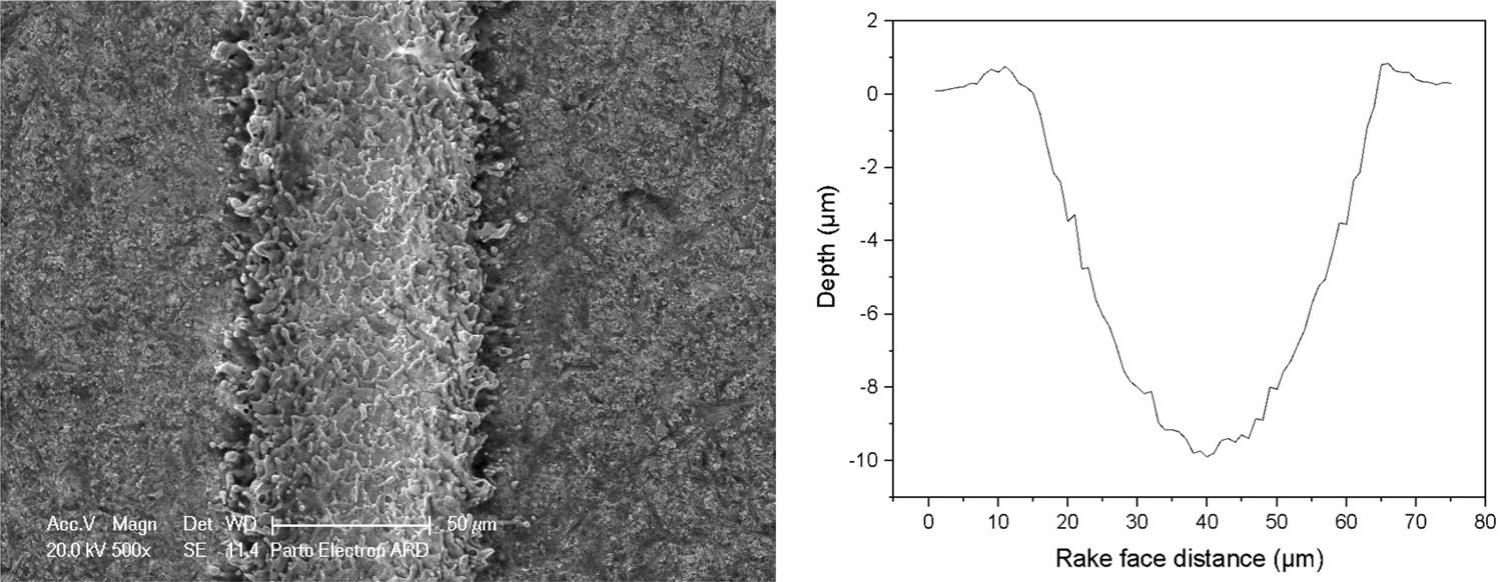
The cross-sectional profile of a micro-groove.

The cutting tools in the experimental tests were cemented carbide CNMA120408 inserts. To engrave microtextures, an Nd: YAG laser from Jinan Xinchu Laser Inc. was employed, operating at a wavelength of 1064 nm, a repetition rate of 20 kHz, and a pulse duration of 10 ns. In order to generate microtextures on the rake face of the tools, the cutting insert was fixed on a computer-controlled translation table, and the laser beam was focused on the rake face perpendicularly and scanned to generate microgrooves. After the laser micro-machining, ultrasonic cleaning was applied to clean the inserts. Following the laser micro-machining, ultrasonic cleaning was performed to ensure the cleanliness of the inserts. In this research, a Fiber laser with a maximum power output of 30 W was utilized for the purpose of texturing the tools. Figure 1 provides a visual representation of the tools in both their plain and textured states, as observed through SEM images. The dimensions of the textures were determined based on a thorough review of relevant literature sources^22,23^, as well as a series of preliminary experiments. Previous research conducted by other scientists has demonstrated that micro-grooves with depths ranging from 10 to 100 μm, widths between 20 and 50 μm, and spacing of 100–300 μm have yielded favorable outcomes in terms of cutting performance. Hence, the selected dimensions for the microgrooves were as follows: a width of 50 μm, a spacing of 150 μm, and a depth of 10 μm. The tool configurations used in this study included linear textures perpendicular to the chip flow direction, linear textures parallel to the chip flow direction, circular textures, and linear cross-hatch textures, referred to as T-Pe, T-Pa, T-Ch, and T-C, respectively. The non-textured plane tool was denoted as T0. Figure 2 provides a depiction of the cross-section profile of a single microgroove generated on the rake face of the carbide insert. The tool with linear textures perpendicular to the chip flow direction, linear textures parallel to the chip flow direction, circular textures, and linear cross-hatch textures were nominated T-Pe, T-Pa, T-Ch, and T-C, respectively, while, the non-textured plane tool was named T0. Figure 2 shows the cross-section profile of an individual microgroove created on the rake face of the carbide insert.

**Figure 3:**
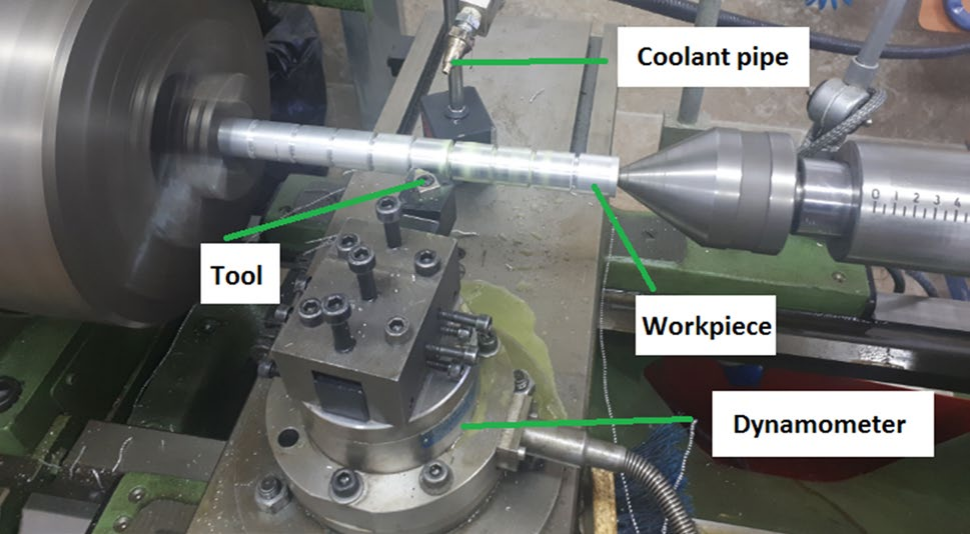
The configuration used for conducting the cutting tests.

The turning experiments were carried out using a TB50NR Lathe equipped with cemented carbide CNMA120408 inserts. Workpieces made of cold-rolled Al 7075 alloy bars with a diameter of 30 mm and a length of 450 mm were employed. The cutting tests were conducted at three cutting speeds (V_c_), 33 m/min, 47 m/min, and 66 m/min, with a fixed depth of cut (a_p_) of 0.75 mm and a feed rate (a_f_) of 0.14 mm/rev. To measure cutting forces, a KISTLER Company turning dynamometer 9272-type with three components was employed. A piezoelectric dynamometer was mounted on the tool post and a tool holder was attached to it. As the cutting operation took place, mechanical forces exerted on the cutting tool were converted into electrical signals by the piezoelectric sensor and then transferred to the charge amplifier. After amplification, the signals were transmitted to the data acquisition system and subsequently transferred to a PC for analysis using the Dyno-Ware software. In every experiment, the PCE-RT Roughness Tester was utilized to measure the surface roughness (Ra). To accomplish this, three specific areas on the machined surface were selected for measurement. The measurements were taken in these regions, and the average of the three measurements was recorded as the Ra value. The tracing velocity and the sampling length were consistently maintained at 0.5 mm/s and 0.8 mm, respectively. The height of the built-up edge was measured using an optical Dino-Lite AM-413ZT microscope with DinoCapture 2.0 software. Tool images were captured, and measurements were taken using calibration tools for precise evaluation of the built-up edge size. SEM analysis was employed to examine the texture patterns on the rake face. The experimental setup for the cutting tests is depicted in Fig. 3.

To create the nanofluid, the process involved introducing CNT particles into an emulsion-type cutting fluid (AMIX: combined ratio with water of 5%). The concentration of CNT particles in the base fluid was set to 1% and 3% based on previous studies on nanofluid applications in machining processes^26, 29, 33–36^. Nanofluids were prepared by dispersing a specific amount of CNTs in the base fluid by using an ultrasonic processor for 6 h generating pulses of 400 W at 24 kHz. This method guarantees the stability of the nanofluid for 24 h^37^. Table 1 provides an overview of the properties of the nanoparticles.

**Table 1:** The characteristics of CNT (Single-Walled Carbon Nanotube) nanoparticles.

Diameter	Length	Purity	Bulk density	Real density	Appearance
1–2 nm	3–8 μm	> 90%	0.17–0.30 g/cm^3^	2.3 g/cm^3^	Black powder

## Results and discussion

### Textured tools

**Figure 4:**
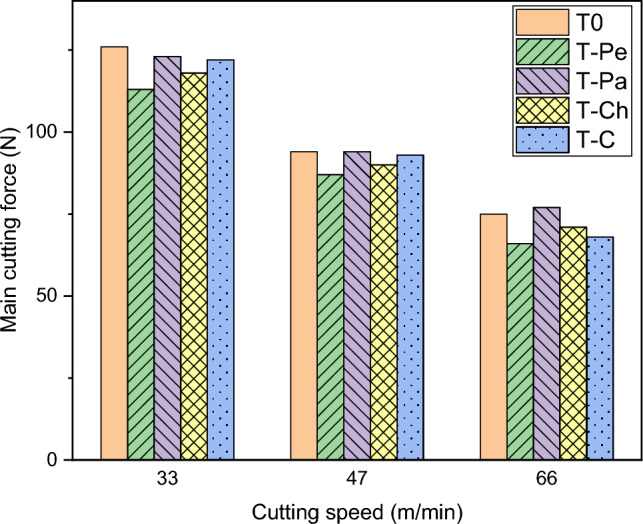
Main cutting force for five types of cutting tools at different cutting speeds.

Figure 4 illustrates the main cutting force observed at various cutting speeds for both non-textured and textured tools. Each bar in the chart represents the average cutting force measured during the turning of Al 7075 alloy. The chart clearly demonstrates that cutting speed exerts a significant influence on the main cutting force. It was observed that as the cutting speed increased, the main cutting force decreased. This phenomenon can be attributed to the thermal softening of the material at higher cutting speeds, resulting in a reduction in shear strength within the shear zone ^38^. Additionally, the shear angle tends to increase with cutting speed. Consequently, the main cutting force can be effectively reduced by increasing the cutting speed during the cutting process, as further discussed below:

The total cutting force, denoted as *F*_*R*_, is determined during the turning process using Eq. (1) ^39,40^:

1 $${F}_{R}={A}_{s}{\tau }_{s}=\frac{{\tau }_{s}{A}_{c}}{sin \varphi }$$

In this equation, *A*_*s*_ represents the shear plane area, *A*_c_ denotes uncut chip cross-section area,  *τ*_s_ signifies the shear strength of the workpiece material, and *φ* represents the shear angle.

The relationship between the main cutting force, denoted as *F*_*c*_, in the turning process and the total cutting force *F*_*R*_ can be expressed by the Eq. (2)^39,40^:

2 $${F}_{c}={F}_{R}sin \left(\beta -\alpha \right)$$

where *β* is the friction angle, and *α* is the rake angle. By substituting Eq. (1) into Eq. (2), we obtain the main cutting force relation expressed in Eq. (3)^39,40^:

3 $${F}_{c}=\frac{{\tau }_{s}{A}_{c}}{sin \varphi }sin \left(\beta -\alpha \right)$$

According to Eq. (3), a decrease in shear strength (*τ*_*s*_) and an increase in shear angle (*φ*) result in a reduction in the main cutting force.

As depicted in Fig. 4, the utilization of textured tools led to a slight reduction in the main cutting force. Among the various textured tools, the T-Pe tool with linear micro-grooves perpendicular to the chip flow direction exhibited the lowest cutting force. The results indicate that, on average, the main cutting force of the T-Pe tool was reduced by 7%, 10%, and 14% at cutting speeds of 33 m/min, 47 m/min, and 66 m/min, respectively, compared to the non-textured tool. The performance of the T-Pe tool in reducing cutting forces was observed to be superior to that of the T-Pa, T-CH, and T-C tools. This can be attributed to the fact that in the T-Pa, T-CH, and T-C tools, there was a higher degree of plastic deformation in the chip material compared to the T-Pe tool, which led to greater adhesion of the work material on the rake face, resulting in higher cutting forces.

Furthermore, the micro-grooves positioned perpendicular to the chip flow direction serve as chip breakers, facilitating the chip's swift and effortless detachment from the rake face. Consequently, the tool-chip contact area is diminished. According to Eq. (4), a decrease in the contact area results in a reduction of the frictional force, thereby leading to a decrease in the total cutting force. As a result, the micro-grooves oriented perpendicular to the chip flow direction exhibited a smaller tool-chip contact area compared to other tools, resulting in a greater reduction in cutting force.

The decrease in the main cutting force when using micro-textured tools can be elucidated as follows:

The frictional force between the chip and the rake face during the turning process can be described by Eqs. (4) and (5)^39,40^:

4 $${F}_{f}={A}_{w}{\tau }_{c}$$

5 $${A}_{w}=l{a}_{w}$$

**Figure 5:**
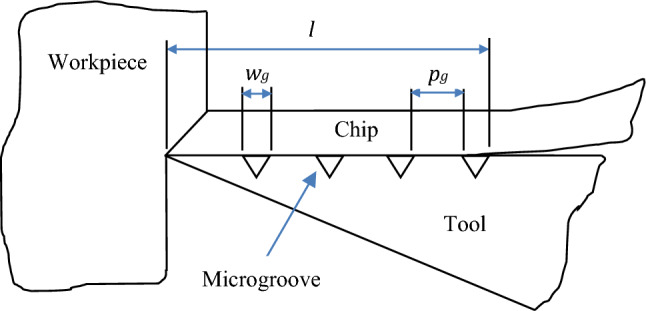
Schematic of tool-chip contact length for a textured tool (*l*_*e*_ = *l−n.w*_*g*_ = *n.p*_*g*_).

In Eq. (4), *A*_w_ represents the contact area between the tool and chip, *τ*_*c*_ denotes the shear strength of the tool-chip interface, *l* represents the contact length between the tool and chip, and *a*_w_ represents the chip width. As illustrated in Fig. 5, the effective contact length of the tool chip can be determined as follows^19,39^:

6 $${l}_{e}=l-n{w}_{g}\approx n{p}_{g}$$

In the Eq. (6), the contact length is represented by *l*, the effective contact length is denoted as *l*_*e*_, *w*_*g*_ signifies the width of the microgrooves, *p*_*g*_ represents the spacing of the microgrooves, and n indicates the number of grooves in the contact area. According to Eq. (6), the effective contact length decreases with the generation of microgrooves on the rake face. Consequently, the contact area (*A*_*w*_) and friction force (*F*_*f*_) decrease. On the other hand, there is a correlation between the main cutting force and the friction force, which can be expressed as follows^39,40^:

7 $${F}_{f}={F}_{R}sin \left(\beta \right)$$

8 $${F}_{c}={F}_{R}cos \left(\beta -\alpha \right)$$

9 $${F}_{c}={F}_{f}\frac{cos \left(\beta -\alpha \right) }{sin \left(\beta \right)}$$

Therefore, it can be inferred that the main cutting force, *F*_*c*_, decreases when the friction force, *F*_*f*_, is reduced.

In the case of the cross-hatch pattern, significant chip deformation occurs as the chip traverses the rake face of the tool. This leads to the bending of the chip towards the micro-grooves. Consequently, the actual contact area between the rake face and chip back increases, diminishing the effectiveness of micro-texturing in reducing forces.

**Figure 6:**
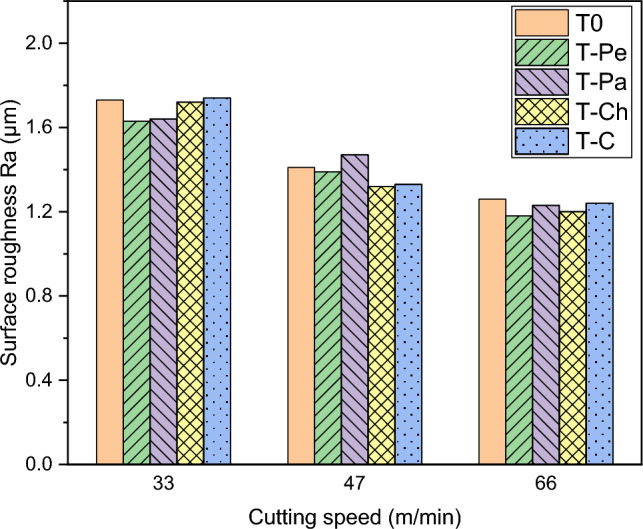
Surface roughness (Ra) for textured and non-textured tools at different cutting speeds.

In Fig. 6, the surface roughness (Ra) of workpieces machined using various textured and non-textured tools is presented. The chart demonstrates that the surface roughness of the machined workpieces improves as the cutting speed increases. This improvement can be attributed to the reduction in built-up edge size and the increased stability of the machining conditions at higher cutting speeds. As a result, the surface finish is enhanced ^40^.

The results indicate that the fabrication of microtextures on the rake face of cemented carbide tools does not have a substantial impact on the surface roughness.

**Figure 7:**
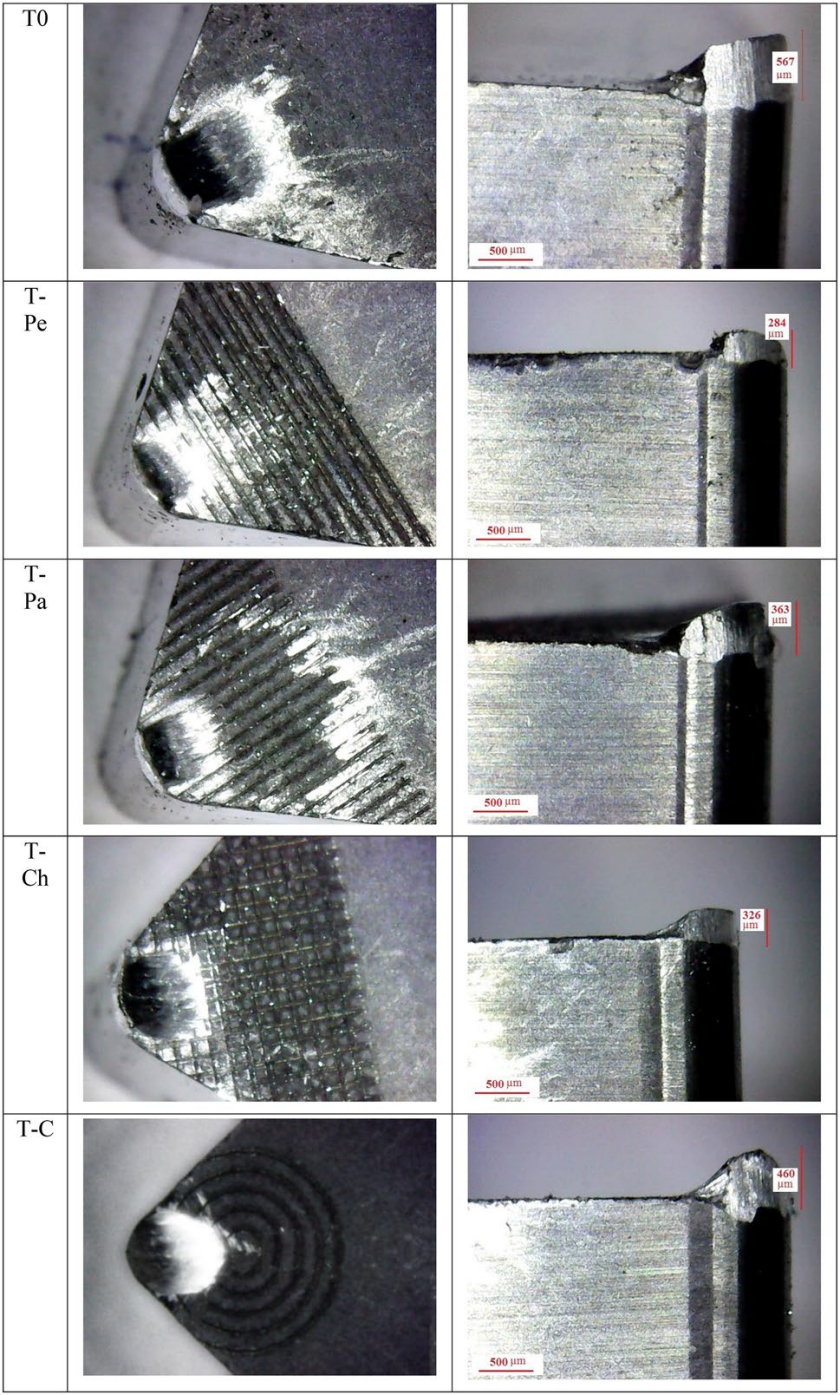
Built up edge height for different tools.

Figure 7 illustrates the size of the built-up edge (BUE) for different tools. It is evident that the textured tools exhibit a lower BUE compared to the non-textured tools. The height of the BUE for the non-textured tool (T0) was measured as 567 μm, whereas it was reduced to 460 μm, 363 μm, 326 μm, and 284 μm for the T-C, T-Pa, T-Ch, and T-Pe tools, respectively. This reduction corresponds to a decrease of 19%, 36%, 43%, and 50%, respectively. Therefore, the use of micro-textured tools results in a reduction in the adhesion of work material on the rake face. As discussed earlier, surface texturing of the rake face reduces the friction force between the tool and chip, leading to decreased heat generation and subsequently minimizing the adhesion of work material on the rake face.

The performance of the cutting process was enhanced by using the T-Pe tool with linear microgrooves arranged perpendicular to the direction of chip flow, as indicated by the outcomes of dry turning tests comparing various textured tools and a conventional tool. To assess the impact of nanofluid lubrication on cutting performance, experimental turning tests were conducted using the chosen tool while employing nanofluid lubrication. The subsequent results are provided below.

### Nanofluid effect

**Figure 8:**
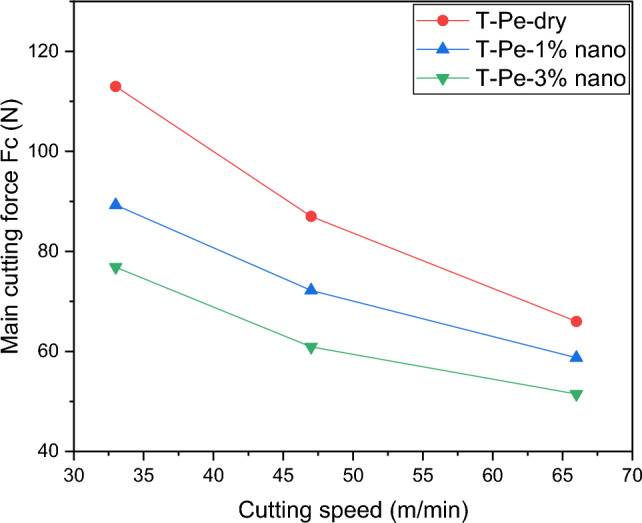
The main cutting force with the cutting speed for different lubrication conditions.

**Figure 9:**
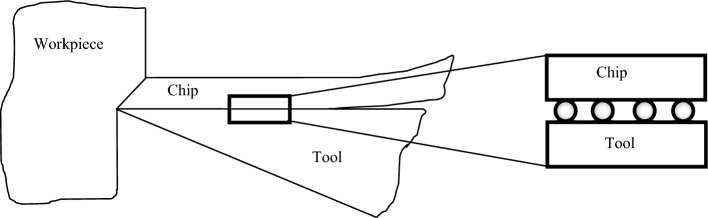
Nano-bearing effect of carbon nanotubes.

Figure 8 illustrates the impact of using CNT-enriched nanofluid lubrication on the main cutting force. The experimental results revealed that compared to dry cutting with the T-Pe textured tool, the main cutting force decreased by up to 21% and 32% when utilizing 1% and 3% CNT nanofluid, respectively. Consequently, an increase in the concentration of nanoparticles improved the lubrication capability of the nanofluid. Figure 9 schematically demonstrates that carbon nanotubes dispersed in the nanofluid penetrate between the tool and chip, functioning as nano-bearings, as discussed in several articles ^41–44^. This alteration in the relative motion between the tool and chip, from sliding to rolling, accounts for the reduction in friction and cutting force. In essence, the decrease in friction and cutting force can be attributed to the nano-bearing effect, which is based on the rolling motion of carbon nanotubes.

**Figure 10:**
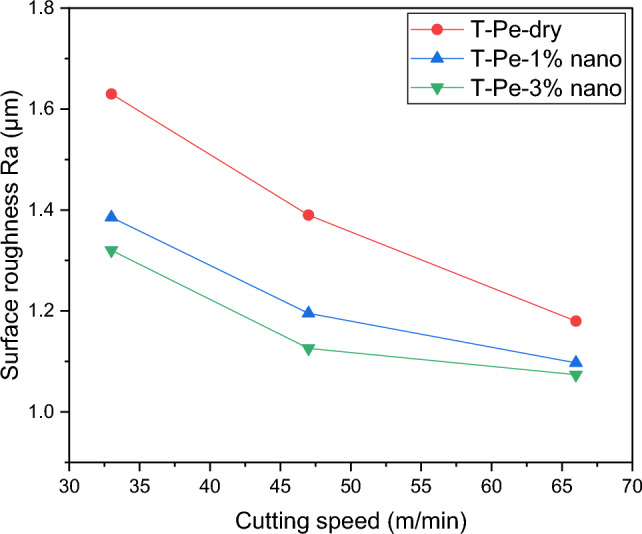
Surface roughness of machined workpiece under different lubricating conditions.

**Figure 11:**
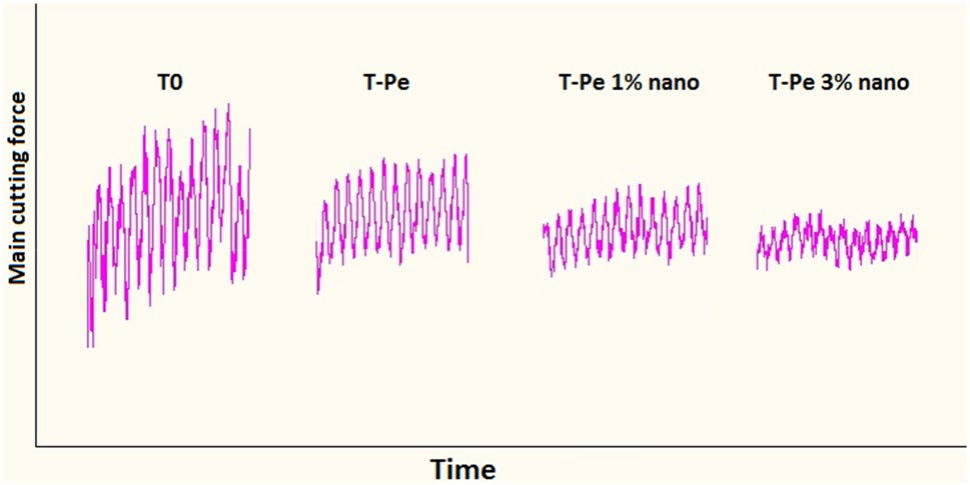
Dynamic main cutting force for different tools at the cutting speed of 66 m/min.

Figure 10 displays the surface roughness (Ra) of machined workpieces using T-Pe textured tools under various lubrication conditions. As previously mentioned, surface texturing on the rake face had an insignificant impact on surface finish. However, when the T-Pe textured tool was utilized with CNT-enriched nanofluid lubrication, an improvement in surface finish was observed. The chart illustrates that Ra was enhanced by 15% and 19% when employing 1% and 3% concentration nanofluids, respectively, compared to dry machining with the T-Pe textured tool. This improvement can be attributed to the stable cutting conditions achieved during the turning process with the T-Pe tool under nanofluid lubrication. Figure 11 exhibits dynamic force profiles for different tools. It is evident from the figure that the fluctuation of cutting force was reduced when using the T-Pe tool with CNT nanofluid lubrication compared to dry conditions. This reduction in fluctuation resulted in a more stable cutting environment, leading to a superior surface finish.

**Figure 12:**
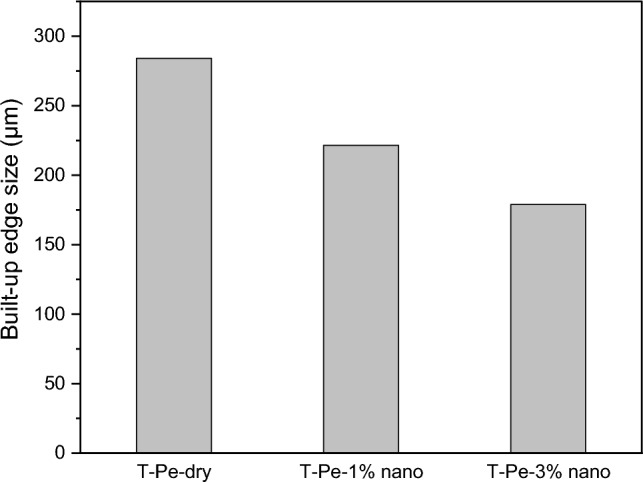
BUE size for different lubrication conditions.

Figure 12 presents the impact of surface texturing and nanofluid lubrication on the size of the built-up edge (BUE). The results demonstrate a significant reduction in BUE size when CNT-enriched nanofluid coolant is used during the turning process with the T-Pe textured cutting tool. As previously mentioned, the addition of CNT nanoparticles to the base coolant enhances the tribological performance of mating surfaces. Consequently, the friction coefficient between the chip and tool decreases, leading to a reduction in friction force at the rake face. This decrease in friction force results in reduced heat generation, which helps mitigate the adhesion of work material to the rake face.

The experimental findings indicate that increasing the nanoparticle concentration from 1 to 3% leads to a greater decrease in BUE size compared to dry cutting with the T-Pe textured tool. Specifically, the decrease in BUE size increased from 22 to 37%. This can be attributed to the influence of nanoparticle concentration on the thermal characteristics of nanofluids. The thermal conductivity (*k*) and convection coefficient (*h*) of nanofluids increases with higher nanoparticle concentrations ^33,34^. Hence, additional heat can be effectively transferred away from the cutting zone, thereby reducing adhesive wear.

The main limitation of the study is the current delivery method of the nanofluid to the cutting zone, which is in the splash mode. We acknowledge that better results could be achieved by designing a more effective nozzle that ensures the efficient delivery of the fluid directly to the cutting area.

## Conclusion

In conclusion, this study aimed to investigate the impact of surface texturing and the application of CNT-enriched nanofluid lubrication on the cutting performance of cemented carbide cutting inserts during the turning of Al 7075 alloy. The results clearly demonstrated that the use of CNT-enriched nanofluid has significant potential in reducing cutting force and surface roughness and minimizing the size of the built-up edge.

Specifically, when comparing the non-textured tool to the application of the T-Pe tool under dry cutting conditions, a decrease of up to 14% in the average main cutting force was observed. Among the investigated textures, the T-Pe texture exhibited superior performance in terms of surface roughness. The implementation of surface textures led to a reduction in friction force, resulting in a decreased height of the built-up edge (BUE). In the absence of lubrication, the height of the BUE was reduced by 19%, 36%, 43%, and 50% for the T-C, T-Pa, T-Ch, and T-Pe tools respectively, in comparison to the non-textured tool.

Furthermore, the experimental findings using the T-Pe textured tool revealed that increasing the concentration of CNT nanoparticles in the base cutting fluid within the range of 1–3% resulted in significant reductions in the main cutting force (Fc) by approximately 21% and 32%, reductions in built-up edge (BUE) sizes of approximately 22% and 37%, and surface roughness reductions of approximately 15% and 19% respectively.

In summary, the findings of this study demonstrate that the creation of appropriate micro textures on the rake face of the turning tool can significantly enhance the machining performance of Al 7075 alloy. Moreover, the use of CNT nanofluid further enhances the performance of the turning process by reducing cutting force, minimizing the size of the built-up edge, and improving surface roughness. These results highlight the potential of surface texturing and the application of CNT-enriched nanofluid lubrication as effective strategies for optimizing cutting processes and achieving improved machining outcomes.

For future studies, it would be beneficial to investigate the online application of ultrasonic probe homogenizer during the turning process to enhance the stability of the nanofluids. This could provide more control and consistency in the lubrication process, leading to further improvements in cutting performance.

## Data Availability

All data generated or analyzed during this study are included in this published article.

## References

[1] Xing Y, Deng J, Wang X, Ehmann K, Cao J (2016). Experimental assessment of laser textured cutting tools in dry cutting of aluminum alloys. J. Manuf. Sci. Eng..

[2] Byrne G, Dornfeld D, Denkena B (2003). Advancing cutting technology. CIRP Ann..

[3] Haddadzade, M., Razfar, M. & Farahnakian, M. Integrating process planning and scheduling for prismatic parts regard to due date. *Int. J. Industrial and Manuf. Engineering* **3**, 248–251 (2009).

[4] Elhami, S., Razfar, M., Farahnakian, M. & Rasti, A. Application of GONNS to predict constrained optimum surface roughness in face milling of high-silicon austenitic stainless steel. *Int. J. Adv. Manuf. Technol.* **66**, 975–986 (2013).

[5] Lutey A (2018). Towards laser-textured antibacterial surfaces. Sci. Rep..

[6] Chetan, Ghosh S, Rao PV (2015). Application of sustainable techniques in metal cutting for enhanced machinability: A review. J. Clean. Prod..

[7] Kharanzhevskiy EV, Ipatov AG, Makarov AV, Gil’mutdinov FZ (2023). Towards eliminating friction and wear in plain bearings operating without lubrication. Sci. Rep..

[8] Gupta MK (2022). Measurement and analysis of machining induced tribological characteristics in dual jet minimum quantity lubrication assisted turning of duplex stainless steel. Measurement.

[9] Danish M (2022). Environmental, technological and economical aspects of cryogenic assisted hard machining operation of inconel 718: A step towards green manufacturing. J. Clean. Prod..

[10] Khani S, Farahnakian M, Razfar MR (2015). Experimental study on hybrid cryogenic and plasma-enhanced turning of 17–4PH stainless steel. Mater. Manuf. Process..

[11] Hasin F (2023). Impact of nanoparticles on vegetable oil as a cutting fluid with fractional ramped analysis. Sci. Rep..

[12] Khanali M, Farahnakian M, Elhami S, Khani S (2022). Tribological properties of vibro-mechanical texturing during face turning processes. Int. J. Lightweight Mater. Manuf..

[13] Baskar, N., V. H. & Sankaran, R. Performance of cutting tool with cross-chevron surface texture filled with green synthesized aluminium oxide nanoparticles. *Sci. Rep*. **9**, 1–9 (2019) 10.1038/s41598-019-54346-0 PMC6882882 31780736

[14] Conradi M, Drnovšek A, Gregorčič P (2018). Wettability and friction control of a stainless steel surface by combining nanosecond laser texturing and adsorption of superhydrophobic nanosilica particles. Sci. Rep..

[15] Niketh S, Samuel GL (2017). Surface texturing for tribology enhancement and its application on drill tool for the sustainable machining of titanium alloy. J. Clean. Prod..

[16] Arumugaprabu V (2018). Performance of surface-textured end-mill insert on AISI 1045 steel. Mater. Manuf. Process..

[17] Sivaiah P, Ajay Kumar GV, Singh MM, Kumar H (2020). Effect of novel hybrid texture tool on turning process performance in MQL machining of Inconel 718 superalloy. Mater. Manuf. Process..

[18] Khani S, Haghighi SS, Razfar MR, Farahnakian M (2021). Optimization of dimensional accuracy in threading process using solid-lubricant embedded textured tools. Mater. Manuf. Process..

[19] Khani S, Razfar MR, Haghighi SS, Farahnakian M (2020). Optimization of microtextured tools parameters in thread turning process of aluminum 7075 aerospace alloy. Mater. Manuf. Process..

[20] Khani S, Shahabi Haghighi S, Razfar MR, Farahnakian M (2021). Improvement of thread turning process using micro-hole textured solid-lubricant embedded tools. Proc. IMechE Part B J. Eng. Manuf..

[21] Xie J, Luo MJ, Wu KK, Yang LF, Li DH (2013). Experimental study on cutting temperature and cutting force in dry turning of titanium alloy using a non-coated micro-grooved tool. Int. J. Mach. Tools Manuf..

[22] Fang Z, Obikawa T (2017). Cooling performance of micro-texture at the tool flank face under high pressure jet coolant assistance. Precis. Eng..

[23] Liu Y (2017). Wear resistance of carbide tools with textured flank-face in dry cutting of green alumina ceramics. Wear.

[24] Santana TD, de Rossi W, Barbosa PA, Bertolete M (2023). Performance of cutting-tool patterns textured via ultrashort laser pulses in the turning of martensitic stainless steel under dry and lubricated conditions. Proc. IMechE Part B J. Eng. Manuf..

[25] Mbambo MC (2020). Thermal conductivity enhancement in gold decorated graphene nanosheets in ethylene glycol based nanofluid. Sci. Rep..

[26] Wang, X. *et al.* Nanofluids application in machining: a comprehensive review. *Int. J. Adv. Manuf. Technol.*, 1–52 (2023).

[27] Prabhu S, Vinayagam B (2010). Nano surface generation of grinding process using carbon nano tubes. Sadhana.

[28] Prabhu S, Vinayagam BK (2012). AFM investigation in grinding process with nanofluids using Taguchi analysis. Int. J. Adv. Manuf. Technol..

[29] Rao, S. N., Satyanarayana, B. & Venkatasubbaiah, K. Experimental estimation of tool wear and cutting temperatures in MQL using cutting fluids with CNT inclusion. *Int. J. Eng. Sci. Technol.* **3**, 2928–2932 (2011).

[30] Sharmin I, Gafur MA, Dhar NR (2020). Preparation and evaluation of a stable CNT-water based nano cutting fluid for machining hard-to-cut material. SN Appl. Sci..

[31] Mahapatra S, Das A, Jena PC, Das SR (2022). Turning of hardened AISI H13 steel with recently developed S3P-AlTiSiN coated carbide tool using MWCNT mixed nanofluid under minimum quantity lubrication. Proc. Inst. Mech. Eng. Part C J. Mech. Eng. Sci..

[32] Pradhan S, Das SR, Jena PC, Dhupal D (2022). Investigations on surface integrity in hard turning of functionally graded specimen under nano fluid assisted minimum quantity lubrication. Adv. Mater. Process. Technol..

[33] Khajehzadeh M, Moradpour J, Razfar MR (2019). Influence of nanolubricant particles’ size on flank wear in hard turning. Mater. Manuf. Process..

[34] Khajehzadeh M, Moradpour J, Razfar MR (2019). Influence of nanofluids application on contact length during hard turning. Mater. Manuf. Process..

[35] Sayuti M, Sarhan AA, Salem F (2014). Novel uses of SiO_2_ nano-lubrication system in hard turning process of hardened steel AISI4140 for less tool wear, surface roughness and oil consumption. J. Clean. Prod..

[36] Sharma AK, Tiwari AK, Dixit AR (2015). Progress of nanofluid application in machining: A review. Mater. Manuf. Process..

[37] Derakhshan MM, Akhavan-Behabadi M (2016). Mixed convection of MWCNT–heat transfer oil nanofluid inside inclined plain and microfin tubes under laminar assisted flow. Int. J. Therm. Sci..

[38] Trent, E. M. & Wright, P. K. Metal cutting. (Butterworth-Heinemann, 2000).

[39] Recent advances in turning with textured cutting tools: A review Journal of Cleaner Production 137701–715 10.1016/j.jclepro.2016.07.138 (2016).

[40] Armarego, E. & Brown, R. H. The machining of metals. 437 (PRENTICE-HALL INC, 1969).

[41] Kamel, B. M., El-Kashif, E., Hoziefa, W., Shiba, M. S. & Elshalakany, A. B. The effect of MWCNTs/GNs hybrid addition on the tribological and rheological properties of lubricating engine oil. *J. Dispersion Science and Technology* **42**, 1811–1819 (2021).

[42] Ramón-Raygoza, E. et al. Development of nanolubricant based on impregnated multilayer graphene for automotive applications: Analysis of tribological properties. *Powder Technology* **302**, 363–371 (2016).

[43] Rawat, S. S., Harsha, A., Das, S. & Deepak, A. P. Effect of CuO and ZnO nano-additives on the tribological performance of paraffin oil–based lithium grease. *Tribology Transactions* **63**, 90–100 (2020).

[44] Srivyas, P. & Charoo, M. A Review on Tribological Characterization of Lubricants with Nano Additives for Automotive Applications. *Tribology in Industry* 40 (2018).

